# Dr. Walter H. Bills

**Published:** 1916-08

**Authors:** 


**Dr. Walter H. Bills,** Buffalo 1870, died at his home in Allegan, Mich., July 10, aged 69.

